# Establishment and Evaluation of an In Vitro System for Biophysical Stimulation of Human Osteoblasts

**DOI:** 10.3390/cells9091995

**Published:** 2020-08-30

**Authors:** Martin Stephan, Julius Zimmermann, Annett Klinder, Franziska Sahm, Ursula van Rienen, Peer W. Kämmerer, Rainer Bader, Anika Jonitz-Heincke

**Affiliations:** 1 Biomechanics and Implant Technology Research Laboratory, Department of Orthopedics, Rostock University Medical Centre, 18057 Rostock, Germany; martin.stephan@med.uni-jena.de (M.S.); annett.klinder@med.uni-rostock.de (A.K.); franziska.sahm@med.uni-rostock.de (F.S.); rainer.bader@med.uni-rostock.de (R.B.); 2 Institute of General Electrical Engineering, University of Rostock, 18059 Rostock, Germany; julius.zimmermann@uni-rostock.de (J.Z.); ursula.van-rienen@uni-rostock.de (U.v.R.); 3 Department Life, Light & Matter, University of Rostock, 18051 Rostock, Germany; 4 Department of Ageing of Individuals and Society, University of Rostock, 18051 Rostock, Germany; 5 Department of Oral and Maxillofacial Surgery, University Medical Centre Mainz, 55131 Mainz, Germany; peer.kaemmerer@unimedizin-mainz.de

**Keywords:** osseointegration, implants, micromotions, electrical stimulation, capacitively coupled electric field, biophysical stimulation, osteoblasts

## Abstract

While several studies investigated the effects of mechanical or electrical stimulation on osseointegration and bone fracture healing, little is known about the molecular and cellular impact of combined biophysical stimulation on peri-implant osseointegration. Therefore, we established an in vitro system, capable of applying shear stress and electric fields simultaneously. Capacitively coupled electric fields were used for electrical stimulation, while roughened Ti6Al4V bodies conducted harmonically oscillating micromotions on collagen scaffolds seeded with human osteoblasts. Different variations of single and combined stimulation were applied for three days, while samples loaded with Ti6Al4V bodies and untreated samples served as control. Metabolic activity, expression of osteogenic markers and bone remodeling markers were investigated. While combined stimulation showed no substantial benefit compared to sole mechanical stimulation, we observed that 25 µm micromotions applied by roughened Ti6Al4V bodies led to a significant increase in gene expression of osteocalcin and tissue inhibitor of metalloprotease 1. Additionally, we found an increase in metabolic activity and expression of bone remodeling markers with reduced procollagen type 1 synthesis after 100 mV_RMS_ electrical stimulation. We were able to trigger specific cellular behaviors using different biophysical stimuli. In future studies, different variations of electrical stimulation will be combined with interfacial micromotions.

## 1. Introduction

Between 2019 and 2050, the number of persons aged 65 or over globally is projected to more than double, indicating that by 2050 one in every four persons in Europe and Northern America could be aged 65 years or over [1]. Following the higher numbers of elderly people, the demand for regenerative medicine is also increasing. This applies especially to medical implants, which can be required for various therapeutic indications such as knee and hip replacement and dentures. Pilz et al. estimated the number of primary hip replacements performed in Germany in 2040 to grow by 27% from 2010, while projection counts were highest for patients aged 60–70 years [2]. Meanwhile the prevalence of dental implants in the United States has increased from 0.7% in 1999–2000 to 5.7% in 2015–2016 and is expected to jump to 17% in 2026 if the trend continues [3]. Crucial for fast functionality, long-term stability, and the survival result of the implants is a process called osseointegration.

Essentially, osseointegration describes the development of a direct structural and functional connection between ordered, living bone and the surface of a load-carrying implant without any apparent soft-tissue intervention between normal bone and the surface of the implant [4,5]. Briefly, blood cells including red cells, platelets, and inflammatory cells migrate into the tissue surrounding the implant and form a fibrin matrix. This matrix acts as a scaffold for the migration of osteogenic cells, which develop into osteoblasts and form osteoid tissue and new woven bone that eventually remodels into lamellar bone [4,6]. Major factors responsible for the failure of peri-implant osteogenesis include a decreased number and/or activity of osteoblasts, increased osteoclastic activity, an abnormal response to systemic and local stimuli, and mechanical stress, and an impaired vascularization of the peri-implant tissue [7]. Since aging impairs angiogenesis [8] and increases the prevalence of osteoporosis [9], osseointegration is reduced in the elderly, resulting in a higher implant failure risk [7]. While factors such as the implant design, the status of the host bone bed and its intrinsic healing potential, as well as biophysical stimulation enhance osseointegration, implant mobility, and excessive micromotion inhibit the recovery process [5,10,11,12,13,14,15]. Hence, optimal implant design and the addition of biophysical stimulation could help to promote bone healing and reduce the risk of implant failure, leading to fewer revision surgeries and higher satisfaction rates among patients [10]. The influence of implant surface and material on the cellular activity of human osteoblasts has been analyzed in previous studies of our working group [16,17,18,19].

Therefore, this study aimed to establish and evaluate an in vitro system for biophysical stimulations on human osteoblasts. While different variations of biophysical stimulation have been investigated, including interface micromotions [20,21,22] and electrical stimulation via direct current [23,24], inductive coupling [25,26,27], and capacitive coupling [25,28,29,30,31,32], the exact molecular and cellular mechanisms by which osseointegration is influenced remain unclear. With a better understanding of these stimulations at a cellular level, the effectiveness of the enhancement of bone regeneration in the clinical setting can be improved. Therefore, we created a system that allowed us to apply capacitively coupled alternating electric fields and harmonically oscillating micromotions on collagen scaffolds seeded with human osteoblasts. The ability to apply mechanical stress via micromotions and electrical stimulation individually and in combination allowed the investigation of each treatment separately as well as their interaction.

## 2. Materials and Methods

### 2.1. Stimulation System for Applying Capacitively Coupled Electrical Fields and Micromotions

**Figure 1:**
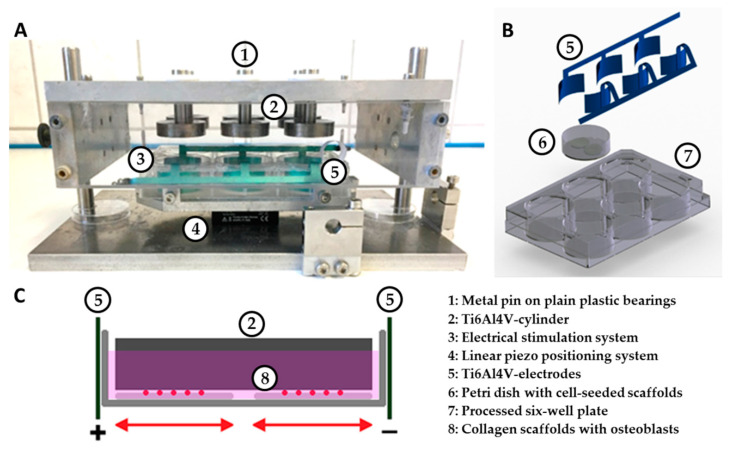
Overview of the components of the stimulation device. (**A**) System for the simultaneous application of capacitively coupled alternating electric fields and harmonically oscillating micromotions on cell-seeded scaffolds. The electrical stimulation system was fixated on top of a linear piezo positioning system using a special rack. The lid of the system holds titanium–aluminum–vanadium alloy (Ti6Al4V) bodies resting on plain plastic bearings on metal pins. (**B**) 3D representation of the individual components of the electrical stimulation system. (**C**) Systematic drawing of two scaffolds with osteoblasts in a petri dish filled with medium. The dish is incorporated into the electrical stimulation system (two Ti6Al4V electrodes), which is moved by the linear positioning system relative to a static Ti6Al4V cylinder.

To apply capacitively coupled electric fields and micromotions individually or in combination to human osteoblasts, an appropriate stimulation system has been established (Figure 1A). To supply osteoblasts with capacitively coupled alternating electrical fields, two electrodes were incorporated into a commercially available six-well cell culture plate made of polystyrene [33]. The material of the electrodes consisted of a titanium–aluminum–vanadium alloy (Ti6Al4V), which is generally known for its excellent corrosion resistance, passivation capacity, and biocompatibility [34]. A 0.5 mm thick, flat Ti6AI4V plate was cut by laser cutting and then anodized using the DOTIZE^®^ process from DOT GmbH, Rostock. The 27 mm wide and 15 mm high electrodes were adapted to the shape of the wells and fitted as tightly as possible to the wall of the well. Three of the electrodes were connected to an electrode strip by a small bridge. To position the collagen scaffolds midway between the electrodes and to enable an optimal sterile work, a sterile petri dish loaded with two cell-seeded scaffolds was inserted into the wells (Figure 1B).

A GX 310 function generator (Metrix, Annecy-le-Vieux, France) was connected to the electrodes to apply a sine wave alternating current with a frequency of 60 kHz, and voltages of either 100 mV_RMS_ or 1 V_RMS_. Frequency and voltage were measured directly on the electrodes using a voltage measuring device (Digital Multimeter VC-960, Voltcraft, Wollerau, Switzerland). Both were varied on the generator until the desired frequency and voltage was displayed. The system ensured that three wells were supplied with the same parameters at the same time. Besides, the setup enabled coupling with the micromotion system, while preventing the possible problems with bio-compatibility of the electrodes and undesired electrochemical processes as well that typically occur with non-capacitive electrical stimulation.

To estimate the effective electric field strength in a single well, a 3D model was generated in COMSOL Multiphysics^®^, v5.3a (COMSOL AB, Stockholm, Sweden). It comprises two electrodes, the culture plate, the petri dish, the cell culture medium (conductivity at 37 °C: 1.6 S/m), and—if applicable—the Ti6Al4V bodies of the micromotion system. The scaffolds were assumed to have the same dielectric properties as the cell culture medium and were thus not explicitly modeled. The electric field was computed using the “electric currents” interface, i.e., in the quasi-static approximation of Maxwell’s equations, at a fixed frequency of 60 kHz. The electric potential at one electrode was set to 0 V while the potential at the other electrode was set to 1.41 V. This corresponded to an applied voltage of 1 V_RMS_. For an applied voltage of 100 mV_RMS_, all values must just be divided by 10. The resulting electric field was evaluated at 1 mm height above the bottom of the dish. This matched to the location of the cells that were to be stimulated.

A two-component chamber system consisting of a base plate and a lid was used to implement micromotions according to Ziebart et al. [20]. A linear piezo positioning system (Physik Instrumente (PI) GmbH & Co. KG, Karlsruhe, Germany) with a rack was attached to the base plate. Three set screws allowed fixating the electrical stimulation system into the rack aligning the system at the exact center of the base plate. The lid consisted of a metal plate as a roof and transparent outer walls. Six holes were drilled into the roof, through which metal pins protruded into the interior of the chamber via plain plastic bearings. Bodies measuring 30 mm in diameter and 8 mm in height were screwed at the end of each pin. The same as the electrodes, the bodies were made of a Ti6Al4V alloy, which is used extensively in dental and orthopedic reconstructive surgery [34]. The undersurface of each cylinder was roughened by DOT GmbH, Rostock, to an average roughness of 20.93 µm (sd 1.98 µm) using corundum beams. Roughness measurements were carried out using a 3D digital laser-scanning microscope (KEYENCE Deutschland GmbH, Neu-Isenburg, Germany). Placing the lid onto the base plate of the chamber enabled the roughened surface of the Ti6Al4V bodies to rest on the collagen scaffolds of the electrical stimulation system, exerting a static pressure load of 527 Pascal (Pa) [20]. Guide rods on the base plate ensured a central positioning of each cylinder into the petri dish. Polystyrene spacers prevented contact between the electrodes of the electrical stimulation system and the lid. Powered, the piezo positioning system moved the scaffolds containing the human osteoblasts horizontally against the roughened surface of the Ti6Al4V-bodies (Figure 1B,C).

By incorporating the electrical stimulation system into the micromotion system, simultaneous stimulation of capacitively coupled alternating electric fields and harmonically oscillating micromotions were implemented in three of the six wells, also allowing a sole stimulation with harmonically oscillating micromotions in the rest. The piezo positioning system was validated using linear variable differential transformers (LVDT, Solartron Metrology, Meerbusch, Germany) clamped into the base plate. Validation was carried out applying peak-to-peak amplitudes of 25 and 100 µm with a frequency of 1 Hz. To determine the deviations of the system, two unsettled scaffolds and 1.7 mL DMEM medium were used as samples. The absolute and percentage deviations, as well as the maximum velocity and acceleration of the micromotion system, are illustrated in Table 1.

### 2.2. Cell Culture and Stimulation

Human osteoblast-like cells were isolated from cancellous bone of the femoral head, donated by patients undergoing primary hip replacement at the Department of Orthopedics of the Rostock University Medical Center. Isolation and cultivation were only implemented if the patient gave their informed consent and were approved by the Local Ethics Committee of the University of Rostock (registration number: A2010-10). Osteoblasts from seven male (mean age 59.4 ± 14.9 years) and eight female (mean age 72.7 ± 10.9 years) donors were used. Donors were chosen randomly. All work was performed under strictly sterile conditions within the Herasafe™ KS biological safety cabinet (Thermo Fisher Scientific, Waltham, MA, USA). Incubation and cultivation of the cell cultures were carried out in an incubator (Binder GmbH, Tuttlingen, Deutschland) at 37 °C and an atmosphere of 5% CO_2_ and 95% air.

To isolate osteoblasts, the protocol of Lochner et al. was used [35]. Osteoblasts were cultivated in a special formulation of Dulbecco’s modified eagle medium (DMEM, w/o calcium) with 10% fetal calf serum (FCS, both: Pan Biotech, Aidenbach, Germany), 1% penicillin/streptomycin (Thermo Scientific, Waltham, MA, USA), 1% amphotericin B (Biochrom GmbH, Berlin, Germany), and 1% N-2-Hydroxyethylpiperazin-N-2-ethane sulfonic acid buffer (HEPES, Gibco^®^, Thermo Fisher Scientific Inc., Waltham, MA, USA). The medium was supplemented with 50 μg/mL L-ascorbic acid (Sigma-Aldrich, Merck, Darmstadt, Germany), 10 mM β-glycerophosphate (Sigma-Aldrich, Merck, Darmstadt, Germany), and 100 nM dexamethasone (Sigma-Aldrich, Merck, Darmstadt, Germany) to support proliferation and maintaining the osteogenic phenotype. To avoid cell contact inhibition, cells were transferred from 80% confluence in a ratio of 1:6 into 75 cm^2^ cell culture flasks.

For scaffold production, a biomatrix from MatriDerm^®^ (MedSkin Solutions Dr. Suwelack AG, Billerbeck, Germany) was used. This open-pore collagen-elastin matrix is primarily used as an acellular dermis replacement and consists of natural bovine collagen types I, II, and V as a fiber template, coated with α-elastin hydrolysate. Collagen-elastin scaffolds allow cell attachment, differentiation, and subsequent mineralization, and thus serves as a suitable framework for the cultivation of osteoblasts [36]. Two MatriDerm^®^ scaffolds measuring 16 mm in diameter and 1 mm in height were fixated side by side to the bottom of a 35 mm Ø petri dish (Thermo Fisher Scientific, Waltham, MA, USA) using biocompatible silicone paste (Korasilon; Kurt Obermeier GmbH & Co. KG, Bad Berleburg, Germany).

The third passage of osteoblasts was cultivated up to 90% confluency and then seeded on the prepared collagen scaffolds. To this end, 200 µL medium containing approximately 100,000 osteoblasts was dropped onto each scaffold. Cell numbers were determined manually using a Thoma hemacytometer. Cells were allowed to adhere for 30 min at room temperature. Afterward, 3 mL of the above-mentioned DMEM, supplemented with 50 μg/mL L-ascorbic acid, 10 mM β-glycerophosphate, and 100 nM dexamethasone, was added. After 24 h of cultivation, 1.3 mL of the cell culture medium was removed, preventing overflow caused by the volume of the Ti6Al4V-bodies. The petri dishes containing two cell-seeded scaffolds were placed into each stimulation system. The systems then were transferred to the incubator. The piezo positioning system was controlled using PIMicroMove (Physik Instrumente (PI) GmbH & Co. KG, Karlsruhe, Germany).

Five different treatment groups were investigated:

1. Culture in the electrical stimulation system (sole stimulation with capacitively coupled alternating electric fields (ES)) with 60 kHz and either 100 mV_RMS_ or 1 V_RMS_.
2. Culture in a conventional six-well plate with Ti6Al4V bodies (stimulation with static pressure load (Load)).
3. Culture in the micromotion system (sole stimulation with harmonically oscillating micromotions (MM)) applying peak-to-peak amplitudes of 25 and 100 µm with a frequency of 1 Hz.
4. Culture in the micromotion system with additional electrical stimulation with 60 kHz and 1 V_RMS_ (simultaneous stimulation with harmonically oscillating micromotions and capacitively coupled alternating electric fields (MM + ES)).
5. Culture in the electrical stimulation system with Ti6Al4V bodies (stimulation with capacitively coupled alternating electric fields and static pressure load (Load + ES)).

In addition to the above-mentioned investigated parameters, tests were initially carried out with the mentioned micromotion amplitudes (25 µm, 100 µm, and also 50 µm) combined with 100 mV_RMS_ electrical stimulation. However, since no significant differences in metabolic activity and gene expression rates were found between these combined tests and samples treated with micromotions alone, these parameter combinations were not pursued further. Untreated samples (untreated control in treatment groups 1 and 2) and samples treated solely with Ti6Al4V-bodies (loaded control in treatment groups 3 to 5) served as control. Stimulations were performed on three consecutive days with a stimulation time of 6 h a day.

### 2.3. Cell Biological and Molecular Methods

#### 2.3.1. Metabolic Activity

The metabolic activity was determined 24 h after the last stimulation interval using the water-soluble tetrazolium salt (WST-1) assay (Roche GmbH, Grenzach-Wyhlen, Germany). Each scaffold was carefully transferred into a 12-well plate and overlaid with a reagent of WST-1 and DMEM in a ratio of 1:10. After an incubation period of 1 h at 37 °C and 5% CO_2_, 100 μL of the color-changed solution was transferred as triplicates into a 96-well plate. The formazan dye formed was quantified using the multimode plate reader Infinite 200 pro (Tecan Group Ltd., Maennedorf, Switzerland) at a wavelength of 450 nm and a reference filter of 630 nm. A blank value was carried along with each series of tests and subtracted from the measured values.

#### 2.3.2. Gene Expression Analysis

The scaffolds used for the WST-1 assay were washed with PBS and digested with 500 µL Collagenase A (Roche, Penzberg, Germany; dissolved in Hanks’ Balanced Salt Solution [Sigma-Aldrich, Merck, Darmstadt, Germany]; pH of 7) for 45 min at 37 °C in an incubation shaker. The isolated osteoblasts were centrifuged for 8 min at 120× *g*. Afterwards, the cell sediment was utilized for RNA isolation using the peqGOLD Total RNA Kit (VWR International GmbH, Darmstadt, Germany) following the manufacturer’s instructions. The purified RNA was eluted in 30 µL sterile RNase-free water (Carl Roth GmbH & Co. KG, Karlsruhe, Germany). RNA concentration was then determined using the plate reader Infinite 200 pro. RNase-free water served as blank.

RT-PCR was used for transcription of messenger RNA (mRNA) into complementary DNA (cDNA) and carried out using the High Capacity cDNA Reverse Transcription Kit (Thermo Fisher Scientific, Waltham, MA, USA). RNA of each sample was dissolved in water treated with diethyl dicarbonate (DEPC) and combined with a mix of dNTPs, random primers, and reverse transcriptase in a ratio of 1:1. RT-PCR then took place in a Thermocycler (Biometra GmbH, Goettingen, Germany). Afterward, the transcribed cDNA was diluted with 20 µL DEPC treated water. To quantify amplified genes, qRT-PCR was carried out in duplicates using the innuMIX qPCR MasterMix SyGreen Kit (Analytik Jena, Jena, Germany). Genes listed in Table 2 were analyzed.

According to instructions, 1 µL of amplificated cDNA was mixed with 9 µL of a master mix containing DEPC-water, forward and reverse primers, and the innuMix MasterMix using a mixing ratio of 6:1:1:10. A total of 1 µL RNase-free water served as a negative control. PCR was performed following the manufacturer’s instructions. The evaluation was conducted using the delta-delta Ct (∆∆Ct) method. Therefore, the relative expression of each target mRNA was normalized to a housekeeping gene according to the equation: ∆Ct = Ct _target gene_ − Ct _housekeeper gene_.

Hypoxanthine guanine phosphoribosyltransferase (HPRT) was used as housekeeping gene. Then, ∆∆Ct was determined by comparing the relative amount of target mRNA of the stimulated sample to the respective control using the equation: ∆∆Ct = ∆Ct _stimulated_ − ∆Ct _control_.

#### 2.3.3. Quantification of Procollagen Type I Synthesis

Type I C-terminal collagen propeptide (CICP) can be useful as a biochemical indicator of collagen production [37]. Therefore, CICP was quantified from the sample’s supernatant using the MicroVue CICP ELISA (Quidel, San Diego, CA, USA). The ELISA was performed according to the manufacturer’s instructions. A standard curve was carried along to determine peptide concentrations within samples. The CICP concentration was quantified using the Tecan reader Infinite pro at a wavelength of 405 nm. Afterwards, the measured concentration was normalized to total protein content. Total protein determination was performed using the Invitrogen Qubit Protein Assay Kit (Thermo Fisher Scientific, Waltham, MA, USA). The assay was carried out according to the manufacturer’s instructions. Provided standards were utilized to create a standard curve, which was then used to quantify the total protein content.

#### 2.3.4. Quantification of ALP Synthesis

Alkaline phosphatase (ALP) is a key marker enzyme for osteoblast activity [38], which is primarily synthesized in the liver and bones of mammals [39]. For ALP quantification, p-nitrophenyl phosphate (pNpp) was used, which is hydrolyzed by ALP. The product can be quantified and used as an indirect measure of the ALP activity of the respective test sample. Therefore, the second scaffold was transferred into a 12-well plate and washed twice using TRIS buffer (50 mM, pH = 8.0). The osteoblasts then were lysed for 10 min at room temperature in distilled water containing 1% Triton X and 1% phenylmethylsulfonyl fluoride (PMSF). Afterwards, lysates were incubated with 1 mM pNpp, 100 mM 2-amino-2-methyl-1,3-propanediol, and 5 mM magnesium chloride in distilled water at a pH of 10. After incubating for 1 h at 37 °C and 5% CO_2_, the reaction was stopped by adding a 2 M sodium hydroxide solution. Subsequently, 100 μL (sixfold determination) of the solution was quantified using the plate reader Infinite Pro at a wavelength of 405 nm. A blank value was carried along with each series of tests and subtracted from the measured values.

### 2.4. Statistical and Graphical Evaluation

The statistical and graphical evaluation was implemented using GraphPad Prism 8 (GraphPad Software Inc., San Diego, CA, USA). Results are presented as box plots showing the median, the 25%- and 75%-quartile, and whiskers for minimum and maximum. All data are shown as percentage change compared to the respective control. Different treatment groups were separated using dashed lines. For better visualization, gene expression was graphically represented as percentage of 2^−∆∆Ct^. Statistical evaluation was performed using the ∆∆Ct values. At least five independent donors were used for each experiment. Depending on the material available, one to five technical replicas were performed. For statistical evaluation, raw data were used for all tests. Statistical significance to the respective control was determined using the Wilcoxon signed-rank test. To compare different treatment groups, the Mann–Whitney U test was performed. The level of significance was set to *p* < 0.05.

## 3. Results

### 3.1. Numerical Simulation of the Electrical Fields

**Figure 2:**
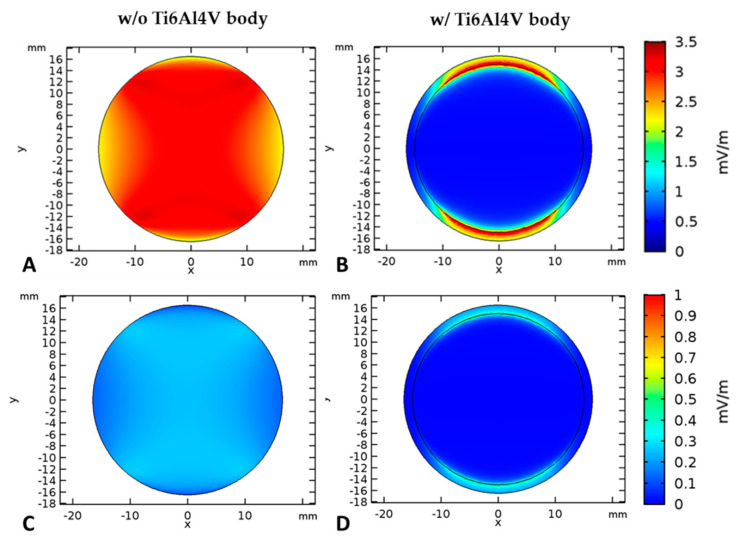
Electric field distribution ((**A**,**B**): input voltage: 1 V_RMS_; (**C**,**D**): input voltage: 100 mV_RMS_) at 1 mm height above the bottom of the chamber (i.e., at cell location) without the Ti6Al4V body (**A**,**C**) and with Ti6Al4V body (**B**,**D**).

The numerical simulation of the electrical field strength revealed both voltage-dependent differences as well as differences with or without the use of TiAl6V4 bodies of the micromotion system (Figure 2). At an input voltage of 1 V_RMS_ the electric field strength is between 2.5 and 3.5 mV/m. Note that this field is significantly influenced by the Ti6Al4V body. In fact, field strengths of 1–3.5 mV/m are only achieved at those edges of the Ti6Al4V body that are directly opposite to the electrodes. Below the Ti6Al4V body, no significant electric field can be observed. In the case of an input voltage of 100 mV_RMS_, the electric field strength is only one tenth compared to that one at 1 V_RMS_. Thus, the electric field reaches a maximum of 0.35 mV/m without Ti6Al4V body. If the cylinder is present, this maximum value can again only be reached at its edges near the electrodes. As a consequence of the obtained simulation results, an input voltage of 1 V_RMS_ was chosen for the combined stimulation (MM + ES) to reach significant field strengths.

### 3.2. Influence of Electrical Stimulation and Micromotions on the Metabolic Activity of Human Osteoblasts

**Figure 3:**
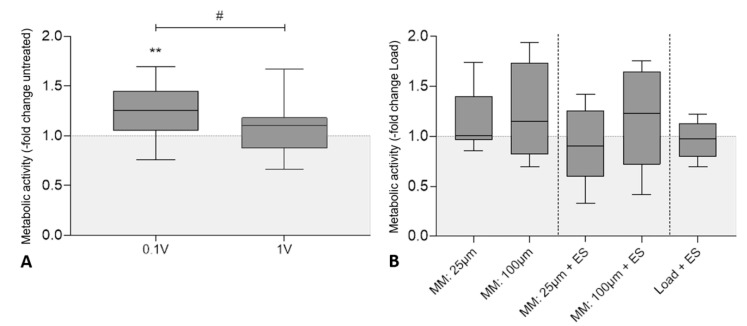
Metabolic activity of human osteoblasts after biophysical stimulation. (**A**) Results of electrical stimulation (ES) with 60 kHz and either 100 mV_RMS_ or 1 V_RMS_. (**B**) Results of micromotions and electrical stimulation with 60 kHz and 1 V_RMS_ (MM + ES) compared to osteoblasts treated solely with micromotions (MM) and osteoblasts treated with titanium bodies and electrical stimulation (Load + ES). Untreated cells with or without titanium bodies served as controls. The metabolic activity was determined colorimetrically by the conversion of tetrazolium salt to formazan. Results are presented as box plots related to the untreated controls (*n* ≥ 6). ** *p* < 0.01 compared to the untreated controls (Wilcoxon’s signed-rank test); # *p* < 0.05 comparison between different voltages (Mann–Whitney U test).

The metabolic activity of human osteoblasts was determined colorimetrically by WST-1 conversion. Figure 3A shows the results after three days of electrical stimulation with 60 kHz and either 100 mV_RMS_ or 1 V_RMS_. Cellular activity was significantly higher in samples treated with 100 mV_RMS_ (Median: 125.5%, *p* = 0.002) compared to the untreated controls while the metabolic activity of cells exposed to 1 V_RMS_ was unaffected. Further, the comparison of both voltages showed a significant difference between the stimulation of 100 mV_RMS_ and 1 V_RMS_ (*p* = 0.017). Load samples showed no significant influence on cell activity levels compared to the untreated control (*p* = 0.964) (Figure S1A).

In Figure 3B the metabolic activity of different treatment groups (micromotions: (MM); micromotions + electrical stimulation: (MM + ES); electrical stimulation plus static pressure load: (Load + ES)) is depicted in comparison to the loaded controls (Load), showing only a slight increase after stimulation with 100 µm micromotions (114.9%) and the combined stimulation of 100 µm micromotions and 1 V_RMS_ electrical stimulation (122.9%) without statistical significance. No further statistically significant difference between the different treatment groups was found.

### 3.3. Influence of Electrical Stimulation and Micromotions on the Expression and Synthesis of Osteogenic Markers in Human Osteoblasts

**Figure 4:**
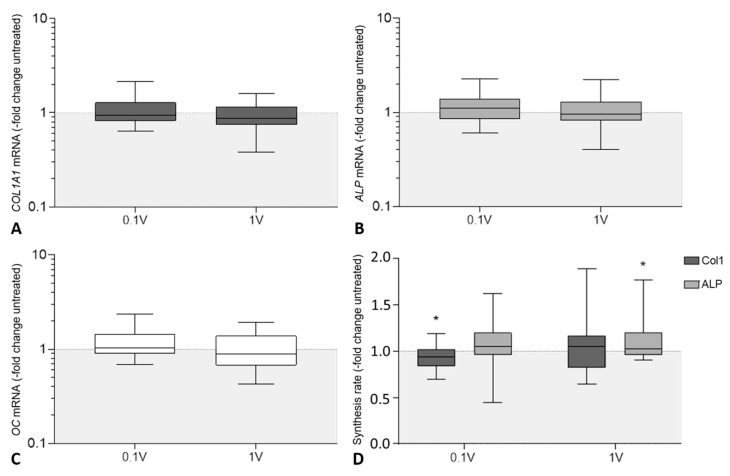
Evaluation of osteogenic differentiation of human osteoblasts following electrical stimulation [ES] with 60 kHz and either 100 mV_RMS_ or 1 V_RMS_. Relative gene expression of osteogenic markers for bone formation: (**A**) collagen type I alpha 1 chain (*COL1A1*), (**B**) alkaline phosphatase (*ALP*) and (**C**) osteocalcin (*OC*). Gene expression levels of osteogenic marker were determined via semi-quantitative polymerase chain reaction (qPCR). Results are presented as boxplots of the percentage of 2^(−ΔΔCt)^ related to untreated controls (*n* ≥ 24). (**D**) Synthesis rates of osteogenic markers procollagen type 1 and alkaline phosphatase (ALP). The release of type I C-terminal collagen propeptide in the cell culture supernatant was determined using ELISA and related to the total protein concentration. The ALP activity was determined colorimetrically by the hydrolysis of p-nitrophenyl phosphate. Results are presented as box plots related to untreated controls (*n* ≥ 15). * *p* < 0.05 compared to the untreated controls (Wilcoxon’s signed-rank test).

Gene expression rates of collagen type 1 alpha 1 chain (*COL1A1*), alkaline phosphatase (*ALP*), and osteocalcin (*OC*) were determined to analyze the effects of electrical stimulation and micromotions on mRNA transcription of osteogenic markers (Figure 4A–C). Consecutively, the amount of procollagen type 1 synthesis rate and the ALP activity were evaluated. Concerning gene expression rates, no significant differences neither between the electrically stimulated cells and the untreated control nor in between the different voltages were detected (Figure 4A–C). Treatment with Ti6Al4V bodies in the loaded control nevertheless resulted in significantly lower *COL1A1* (62.2%, *p* < 0.001) and *ALP* (74.0%, *p* = 0.003) mRNA transcripts, while also showing decreased *OC* gene expression rates (80.1%, *p* = 0.315) without statistical significance compared to the untreated control (Figure S1B).

With regard to protein levels, a reduced procollagen type 1 propeptide content was found in the cell culture supernatants after treatment with 100 mV_RMS_ (93.9%, *p* = 0.048 compared to untreated controls; Figure 4D). Stimulation with 1 V_RMS_ led to an unchanged protein release. In contrast, ALP activity was increased after electrical stimulation, reaching significance following treatment with 1 V_RMS_ (*p* = 0.036) (Figure 4D). In accordance with gene expression rates, procollagen type 1 synthesis rate (81.2%, *p* < 0.001) and ALP activity (70.1%, *p* < 0.001) were significantly decreased after treatment with titanium bodies compared to the untreated control (Figure S1C).

**Figure 5:**
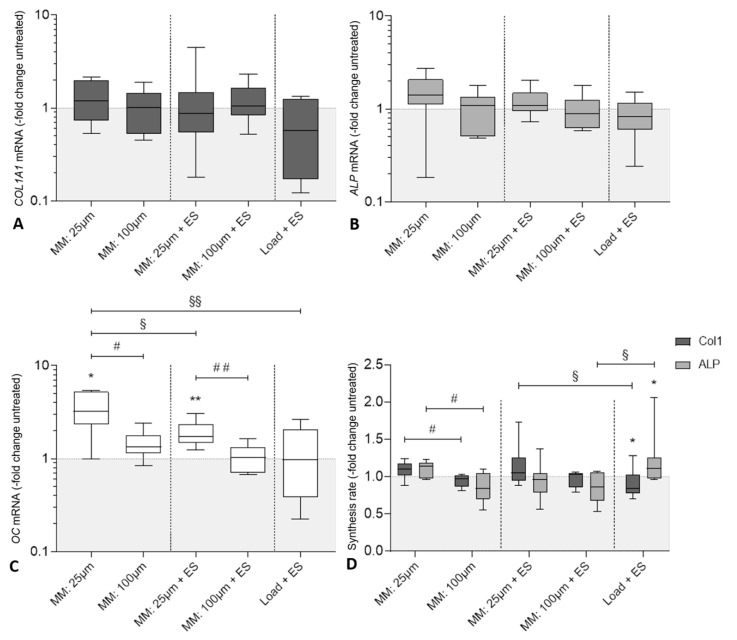
Evaluation of osteogenic differentiation of human osteoblasts following biophysical stimulation. Relative gene expression of osteogenic markers for bone formation: (**A**) collagen type I alpha 1 chain (*COL1A1*), (**B**) alkaline phosphatase (*ALP*), and (**C**) osteocalcin (*OC*) in human osteoblasts after treatment with micromotions and electrical stimulation (MM + ES) compared to osteoblasts treated solely with micromotions (MM) and osteoblasts treated with titanium bodies and electrical stimulation (Load + ES). Gene expression levels of *COL1A1*, *ALP*, and *OC* were determined via semi-quantitative polymerase chain reaction (qPCR). Results are presented as boxplots of the percentage of 2^(−ΔΔCt)^ related to loaded controls (*n* ≥ 6). (**D**) Synthesis rates of osteogenic markers procollagen type 1 and alkaline phosphatase (ALP) in human osteoblasts. The release of type I C-terminal collagen propeptide in the cell culture supernatant was determined using ELISA and related to the total protein concentration. The ALP activity was determined colorimetrically by the hydrolysis of p-nitrophenyl phosphate. Results are presented as box plots related to the loaded controls (*n* ≥ 6). * *p* < 0.05, ** *p* < 0.01 compared to the loaded controls (Wilcoxon’s signed-rank test); ^#^ *p* < 0.05, ^##^ *p* < 0.01 comparison between different parameters (Mann–Whitney U test); ^§^ *p* < 0.05, ^§§^ *p* < 0.01 comparison between different treatment groups (Mann–Whitney U test).

In Figure 5A–C gene expression analysis following electrical stimulation and micromotions is shown in comparison to the loaded control. Although transcription of *COL1A1* and *ALP* was not significantly affected in all stimulation groups, a tendency of increased gene expression rates was found after treatment with 25 µm micromotions. However, compared to the loaded controls, expression of *OC* mRNA was more than tripled (322.9%, *p* = 0.0156) after stimulation with 25 µm micromotions, while almost doubled after the combined stimulation with 25 µm micromotions and 1 V_RMS_ electrical stimulation (171.5%, *p* = 0.008). Further, the comparison within each treatment group showed favorable results in both single (*p* = 0.02) and combined (*p* = 0.008) 25 µm micromotion stimulation compared to treatment with 100 µm micromotions. Comparing the different treatment groups, a higher *OC* gene expression was found after treatment with 25 µm micromotions compared to the combined stimulation of 25 µm micromotions and 1 V_RMS_ electrical stimulation (*p* = 0.027), however, the combined treatment still showed significantly higher gene expression *OC* than electrical stimulation alone (*p* = 0.039; Figure 5C). 

Following gene expression rates, a slight, but not significant upregulated protein release of procollagen type 1 (110%, *p* = 0.078) and ALP activity (114.4%, *p* = 0,148) were observed after treatment with 25 µm micromotions (Figure 5D) compared to the loaded controls. While procollagen type 1 synthesis significantly decreased after treatment with 1 V_RMS_ electrical stimulation in loaded samples (84.2%, *p* = 0.02), ALP activity was significantly increased (110.0%, *p* = 0.025) compared to the loaded control. After treatment with 25 µm micromotions, protein and activity levels were significantly enhanced compared to 100 µm micromotions (*p* = 0.02 for both markers). Evaluating the different treatment groups, procollagen type 1 synthesis was significantly higher following combined stimulation with 25 µm micromotions and 1 V_RMS_ electrical stimulation in comparison to treatment with titanium bodies and electrical stimulation (*p* = 0.021). ALP activity significantly decreased after the combined stimulation with 100 µm micromotions and 1 V_RMS_ electrical stimulation compared to loaded samples treated solely with electrical stimulation (*p* = 0.014).

### 3.4. Influence of Electrical Stimulation and Micromotions on Bone Remodeling

**Figure 6:**
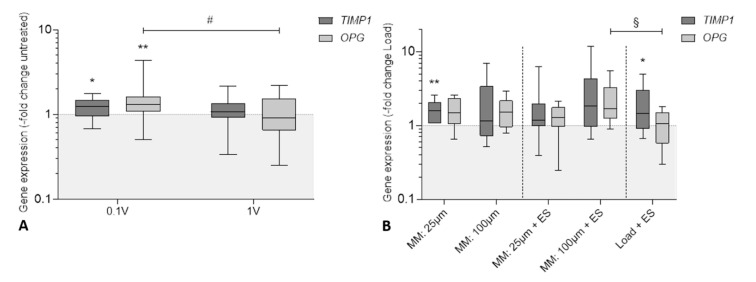
Relative gene expression of genes responsible for bone remodeling: tissue inhibitor of metalloproteinase 1 (*TIMP1*) and osteoprotegerin (*OPG*) in human osteoblasts after biophysical stimulation. (**A**) Results of electrical stimulation (ES) with 60 kHz and either 100 mV_RMS_ or 1 V_RMS_. (**B**) Results of micromotions and electrical stimulation (MM + ES) compared to osteoblasts treated solely with micromotions (MM) and osteoblasts treated with titanium bodies and electrical stimulation (Load + ES). Gene expression levels of *TIMP1* and *OPG* were determined via semi-quantitative polymerase chain reaction (qPCR). Results are presented as boxplots of the percentage of 2^(−ΔΔCt)^ related to the untreated controls (*n* ≥ 6). * *p* < 0.05; ** *p* < 0.01 compared to the untreated controls (Wilcoxon’s signed-rank test); ^#^ *p* < 0.05 comparison between different voltages (Mann–Whitney U test); ^§^ *p* < 0.05 comparison between different treatment groups (Mann–Whitney U test).

Transcripts of matrix metalloproteinase-1 (*MMP1*), osteoprotegerin (*OPG*), receptor activator of nuclear factor kappa-Β ligand (*RANKL*), and tissue inhibitor of metalloproteinase 1 (*TIMP1*) were determined to evaluate the influence of electrical stimulation and micromotions on bone remodeling. However, *RANKL* and *MMP1* mRNA were undetectable in treated and untreated samples since Ct values were higher than the threshold of 30 cycles. Therefore, both markers were excluded from this study. Results after electrical stimulation are visualized in Figure 6A. *TIMP1* (125.1%, *p* = 0.017) and *OPG* (130.8%, *p* = 0.005) mRNA were significantly upregulated after treatment with 100 mV_RMS_ compared to the untreated control. However, both bone remodeling markers were not significantly affected following treatment with 1 V_RMS_. Electrical stimulation with 100 mV_RMS_ also enhanced *OPG* transcripts compared to 1 V_RMS_ (*p* = 0.043). The application of a static pressure load via Ti6Al4V bodies led to a significant upregulation in *TIMP1* gene expression rates (187.3%, *p* < 0.001) compared to the untreated control, while the transcription of *OPG* mRNA was not significantly affected (*p* = 0.235) by the loading (Figure S1D).

Evaluating the expression of both bone remodeling markers following micromotions and electrical stimulation (Figure 6B), *TIMP1* mRNA was upregulated in all stimulation groups, reaching significance when treated with 25 µm micromotions (*p* = 0.008) and 1 V_RMS_ electrical stimulation in loaded samples (*p* = 0.045) compared to the loaded controls. Additionally, enhanced *OPG* transcripts were detectable after treatment with 25 and 100 µm in both single and combined stimulation, without reaching statistical significance. Analyzing the different treatment groups, *OPG* expression was significantly higher after combined stimulation with 100 µm micromotions and 1 V_RMS_ electrical stimulation in comparison to treatment with titanium bodies and electrical stimulation (*p* = 0.042).

## 4. Discussion

While both mechanical and electrical stimulation have been applied widely in bone fracture treatment or bone tissue construction, the effect of combined mechanical and electrical stimulation on osteoblasts or bone tissue has not been deeply studied, although providing a more actual simulation of the in vivo environment [40]. Therefore, the aim of this study was to establish an in vitro system, capable of applying both mechanical stress via micromotions and electrical stimulation on scaffolds seeded with human osteoblasts. Doing so, we were able to further investigate the molecular and cellular impact of biophysical stimulation on peri-implant osseointegration.

The used stimulation parameters for electrical stimulation (voltages) [33] and interface micromotions (peak-to-peak amplitudes, frequency, waveform, and stimulation time) [20] have been selected based on former investigations in our working group. Krueger et al. [33] showed increased synthesis rates of collagen II and glycosaminoglycans in human chondrocytes following electrical stimulation with 100 mV_RMS_ at a utility frequency of 1 kHz using the described electrical stimulation system. To adapt parameters of electrical stimulation to osteoblasts, a study conducted by Brighton et al. [30] was consulted. They found a significant increase in proliferation after the application of sine wave 60 kHz capacitively coupled electrical fields on newborn rat calvarial bone cells when the signal was applied continuously for 6 h. Since Brighton and his team implemented the utility frequency of 60 kHz for capacitively coupled electrical stimulation, this frequency has been used in several other studies [41,42]. The micromotion system was first described by Ziebart et al. [20], who found an increase in metabolic activity and osteocalcin expression level of human osteoblasts after treatment with 25 µm micromotions via plain Ti6Al4V bodies. For better representation of the clinical setting at the bone–implant interface, rough surfaces were used in this present study, imitating the surface of cementless implants after initial implant fixation. While previous in vitro studies have broadly investigated either osteoblastic cell lines like MG-63 or primary cells from different species, we used isolated human osteoblasts for the experiments. The main advantage of primary human cells is their clinical applicability since cell-line osteoblasts and other animal cell sources do not fully reflect the behavior of primary cells. Nonetheless, human isolated osteoblasts represent a heterogeneous cell population, therefore cell response might differ with donor age, location of harvesting, and harvesting method [26,43]. Additionally, 3D collagen scaffolds were used for cell cultivation rather than cell monolayer cultures. Their biomimetic nature has been shown to improve cell adhesion and osteogenic differentiation while accomplishing a more accurate imitation of the natural cell environment [44].

In 1990 Brighton et al. evaluated the efficacy of different electrical field strengths ranging from 0.01 to 20 mV/cm for different patterns of a 60 kHz sine wave. Based on their results and upon comparison to stimulation with 0.0001 mV/cm and 10 Hz, Brighton et al. concluded that proper field strengths ranged from 0.1 to 20 mV/cm [30]. However, in our study the metabolic activity, which was also interpreted as a proliferation marker, was significantly higher after the electrical stimulation with 100 mV_RMS_, leading to an electrical field strength of approximately 0.003 mV/cm in the numerical simulation. The cellular activity was increased, not only compared to the untreated control, but also to samples treated with a 10 times higher voltage of 1 V_RMS_, implying that the lower electrical field strength of 0.003 mV/cm is more effective in stimulating cell proliferation than the higher electrical field strength of 0.03 mV/cm. In another study, Fitzsimmons et al. subjected chick calvarial bone cells to an even lower capacitively coupled 10 Hz sinusoidal electrical field of 0.0001 mV/cm and found an increase in [^3^H]-thymidine incorporation, indicating higher proliferation [30,45]. In their study, Brighton et al. also found that ALP activity was significantly increased after stimulation with 0.1 mV/cm at 60 kHz and heightened after stimulation with 0.01 mV/cm [30]. This was reflected by our observation since ALP activity was significantly enhanced after electrical stimulation with 0.03 mV/cm compared to the untreated control. However, direct comparison is challenging due to differing proliferation and differentiation measuring tools, stimulation setups, application times, and cell lines. While *COL1A1*, *ALP*, and *OC* transcription were not affected by the electrical stimulation, *TIMP1* and *OPG* gene expression were significantly increased after treatment with 100 mV_RMS_. These findings indicate a voltage-dependent alteration of bone remodeling, a hypothesis, which is also supported by a study conducted by Rubin et al. [46]. They found that bone resorption can be prevented by the exogenous application of electric fields below 0.01 mV/cm, when induced at frequencies between 50 and 150 Hz for 1 h/day using an in vivo model of osteopenia. Further investigations will clarify how the use of different field strengths and frequencies can be utilized to promote bone remodeling and prevent excessive bone resorption. As in most compared studies, calculation was used to constitute the electrical field strength, representing only a rough estimate of the actual field strength.

Analyzing the impact of pressure and micromotions on human osteoblasts, both metabolic activity and *OC* gene expression were not significantly affected by treatment with a static pressure load of 527 Pa conducted by the Ti6Al4V cylinders, although *OC* expression rates were slightly downregulated. *Col1A1* and *ALP* transcripts, as well as protein and activity levels, however, were significantly reduced in loaded samples. Similar findings have been described by Pioletti et al. [21] after the application of a comparable pressure load of 500 Pa to MG-63 osteoblast-like cells. They found that cell viability was not affected by loading, while load alone was more potent to downregulate the tested genes (*COL1A1*, *COL1A2*, *OC*, and Osteonectin) than load with micromotion. Nevertheless, in our study, *TIMP1* mRNA transcripts were significantly higher in loaded samples compared to the untreated control and *MMP1* gene expression was undetectable, indicating that bone matrix degradation could be inhibited [47]. In a previous study, with the same design as in the current study, Ziebart et al. [20] described an increase in metabolic activity after stimulation with 25 µm sine micromotions, while Col 1 protein synthesis and gene expression were decreased and ALP activity was not significantly affected. However, our results showed a different response to stimulation. While the metabolic activity was not significantly affected by micromotions in the present study, procollagen type 1 protein synthesis rate and ALP activity were significantly higher after stimulation with 25 µm peak-to-peak amplitudes compared to 100 µm. Additionally, *COL1A1* and *ALP* gene expression rates were also increased. This might indicate that cell proliferation and differentiation can be influenced by the surface applying the micromotions, resulting in different amounts of shear stress and cell adherence [5,48]. Although it must be noted that Ziebart et al. [20] did not normalize procollagen type 1 concentrations to total protein content. Therefore, results might differ. Future experiments will establish whether different surface designs are able to trigger specific cellular behavior. Concurrent with previous findings of our working group *OC* gene expression was significantly increased after treatment with 25 µm micromotions compared to the loaded control [20]. Furthermore, *OC* mRNA transcripts were significantly upregulated in comparison to stimulation with 100 µm micromotions in both single and combined stimulation, indicating more advanced osteogenic differentiation. However, since we have so far only performed a three-day stimulation, we have focused on the osteoblastic calcification capacity only by analyzing the induction of osteocalcin on the mRNA level. As this osteogenic marker was significantly induced by micromotions, we will investigate the protein production of this biomarker in the cell culture supernatant in further studies with longer stimulation periods.

In line with our observations, a consensus in the literature revealed sufficient osseointegration in the presence of interface micromotions of up to 30 µm, while displacements larger than 40 µm seemed to compromise or inhibit the incorporation of non-vital components into living bone [5,12,13,14,15,20]. Further, Nishioka et al. found an increase in ALP, collagenous protein synthesis, and DNA synthesis after the application of mechanical stress to osteoblast-like cells [49]. Interestingly, not only bone formation but also bone resorption seemed to be affected by the application of mechanical stress implemented by micromotions in our study as *TIMP1* mRNA was significantly upregulated after treatment with 25 µm micromotions compared to the loaded control indicating an influence of mechanical stress on bone remodeling processes.

We initially assumed that the combination of different biophysical stimuli could further enhance osteogenic proliferation and differentiation as shown in other studies [40,50]. However, our results indicate that a combination of micromotions and electrical stimulation using capacitively coupled alternating electric fields is not superior to sole stimulation performed by micromotion and can even lead to significantly lower osteocalcin expressions. This might be due to major reasons: first, the electric field is diminished almost to 0 mV/m by the Ti6Al4V bodies, as seen in the numerical simulation. Solely at the edges of the bodies do field enhancements lead to approximately the same field strengths as without a titanium body. Therefore, it can be assumed that only a limited number of osteoblasts are supplied with substantial field strength. The experimental setup revealed that the capacitively coupled electrical fields were predominantly shielded by the conductive surface of the Ti6Al4V bodies. An increase in the input voltages would therefore not be beneficial for the cells located directly below the titanium body. We are aware that this fact is a clear limitation, but we can deduct from this finding that the capacitive coupling of an electric field in combination with a conductive metallic implant might not increase osseointegration in the clinical setting as the implant surface would shield the electrical fields for the cells at the implant periphery. For this purpose, a different arrangement will have to be realized, e.g., the implant itself represents the electrostimulative unit. Such an experimental arrangement would also be conceivable as an experimental setup in future investigations.

Nevertheless, our present data show that the electrical stimulation of loaded samples with 1 V_RMS_ led to significantly higher ALP activity and reduced procollagen type 1 protein levels compared to the loaded control, similar to our findings following the electrical stimulation with 100 mV_RMS_. Additionally, *TIMP1* gene expression was increased after the treatment with Ti6Al4V bodies and 1 V_RMS_ electrical stimulation compared to the loaded control.

A second reason could be attributed to the piezo effect. When stimulating with micromotions, a voltage is generated due to the piezoelectric effect of the scaffolds’ collagen matrix [51]. The origin of this effect, which was first described for collagen by Fukada et al. in 1967, is supposed to be due to the polarization or displacement of hydrogen bonds being formed in the polypeptide chains of collagen crystals and appears when shearing force is applied to the collagen fibers [52,53]. The extent to which these voltages interact with the field of electrical stimulation must be validated in further experiments. Another explanation might be due to overstimulation of the osteoblasts. However, an overstimulation through the different electrical fields is rather unlikely considering the low electric field strengths compared to other working groups, using inductive [26,41] or capacitive coupling [28,29,30,31,32], and the overall reduction of the electrical field by the Ti6Al4V bodies.

In our study, we were not able to detect *MMP1* and *RANKL* transcripts in amounts high enough for analysis. This must be considered, when interpreting the bone remodeling markers *TIMP1* and *OPG*, whereas bone formation and resorption is supposedly regulated by a balance between active TIMPs and MMPs [47] as well as OPG and RANKL [54]. Moreover, different cell types are involved in this process limiting the outcome of this study. Therefore, the influence of biophysical stimulation on osteoclastic cells as well as co-cultures with osteoblasts and osteoclasts are subjects of future studies. This work aimed to analyze the impact and possible interactions between the application of mechanical stress and electric fields. Therefore, only two different parameters were selected for each biophysical stimulus, putting the emphasis on the mode of stimulation. However, due to the substantial reduction of the electric field caused by the Ti6Al4V bodies only the application of 1 V_RMS_ was analyzed for combined stimulation and electrical stimulation in loaded samples. In future experiments, further parameter configuration can be investigated. Nevertheless, considering the necessity of remarkably high energy input to form electric fields comparable to direct stimulation, we recommend the establishment of different variations of electrical stimulation for future investigations, such as semi capacitive and inductive stimulation.

## 5. Conclusions

Our results show that metabolic activity, protein synthesis, and gene expression can be significantly influenced by the application of low capacitively coupled electric fields. We also found that mechanical stress of 25 µm micromotions conducted by the roughened surface of the Ti6Al4V bodies led to significantly higher amounts of *OC* and *TIMP1* gene expression, while procollagen type 1 synthesis rate and ALP activity were also significantly increased compared to 100 µm micromotions. Furthermore, we were able to implement a system that enables the investigation of human osteoblasts after the combined biophysical stimulation of capacitively coupled alternating electric fields and harmonically oscillating micromotions. In future studies, different variations of electrical stimulation such as semi capacitive and inductive stimulation will be validated in combination with interfacial micromotion. Investigations on capacitive coupling without the interference of titanium bodies should also be realized.

## Figures and Tables

**Table 1:** Deviation, maximum velocity, and acceleration in a micromotion system with two scaffolds and 1.7 mL DMEM medium.

Peak-to-Peak Amplitudes	25 µm		100 µm	
	Absolute	Percentage	Absolute	Percentage
Deviation	1.5 µm	6.0%	3.25 µm	3.25%
Maximum velocity	73.8 µm/s		303.9 µm/s	
Maximum acceleration	463.9 µm/s^2^		1909.8 µm/s^2^	

**Table 2:** Genes and primer sequences (purchased from Sigma-Aldrich (Merck), Darmstadt, Germany) analyzed using quantitative Real-Time Polymerase Chain Reaction.

Gene	Sequence
Alkaline phosphatase (ALP)	For: 5′-CATTGTGACCACCACGAGAG-3′ Rev: 5′-CCATGATCACGTCAATGTCC-3′
Collagen type I alpha 1 chain (COL1A1)	For: 5′-ACGAAGACATCCCACCAATC-3′ Rev: 5′-AGATCACGTCATCGCACAAC-3′
Hypoxanthine-guanine phosphoribosyl transferase (HPRT)	For: 5′-CCCTGGCGTCGTGATTAGTG-3′ Rev: 5′-TCGAGCAAGACGTTCAGTCC-3′
Matrix metalloproteinase 1 (MMP1)	For: 5′-AGAGCAGATGTGGACCATGC-3′ Rev: 5′-TCCCGATGATCTCCCCTGAC-3′
Osteocalcin (OC)	For: 5′-TCAGCCAACTCGTCACAGTC-3′ Rev: 5′-GGTGCAGCCTTTGTGTCC-3′
Osteoprotegerin (OPG)	For: 5′-AGGCGATACTTCCTGTTGCC-3′ Rev: 5′-GATGTCCAGAAACACGAGCG-3′
Receptor activator of nuclear factor-kappa-Β ligand (RANKL)	For: 5′-TCTTCTATTTCAGAGCGCAGATGG-3′ Rev: 5′-CTGATGTGCTGTGATCCAACG-3′
Tissue inhibitor of metalloproteinase 1 (TIMP1)	For: 5′-ATTGCTGGAAAACTGCAGGATG-3′ Rev: 5′-GTCCACAAGCAATGAGTGCC-3′

## Supplementary Materials

The following are available online at https://www.mdpi.com/2073-4409/9/9/1995/s1, Figure S1: Influence of a static pressure load of 527 Pa conducted by titanium bodies (load) on osteoblastic viability (**A**) and expression of osteogenic markers (**B**–**D**). Human osteoblasts were seeded on collagen scaffolds and loaded with Ti6Al4V bodies for three days. Untreated cells served as control. (**A**) The metabolic activity was determined colorimetrically by the conversion of tetrazolium salt to formazan (WST-1, *n* = 23). (**B**) Synthesis rates of procollagen type 1 and alkaline phosphatase (ALP) (untreated cells served as control). The release of procollagen type 1 propeptide in the cell culture supernatant was determined using ELISA and related to the total protein concentration. The ALP activity was determined colorimetrically by the hydrolysis of p-nitrophenyl phosphate (both: *n* ≥ 21). (**C**,**D**) Relative gene expression of osteogenic markers for bone formation: collagen type I alpha 1 chain (COL1A1), alkaline phosphatase (ALP) and osteocalcin (OC) and bone remodeling: tissue inhibitor of metalloproteinase 1 (TIMP1) and osteoprotegerin (OPG). Gene expression of *COL1A1*, *ALP, OC* (all in Figure B), *TIMP1,* and *OPG* (both in Figure C) was determined via semi-quantitative polymerase chain reaction (qPCR). Results are presented as the percentage of 2^(−ΔΔCt)^ related to the untreated controls (*n* = 23). All data are presented as box plots related to the untreated controls. ** *p* < 0.01; *** *p* < 0.001 compared to the untreated controls (Wilcoxon’s signed-rank test).
