# Supplementary material for: Establishment and Evaluation of an In Vitro System for Biophysical Stimulation of Human Osteoblasts
Source: Cells. 2020 Aug 30;9(9):1995. doi: 10.3390/cells9091995 (PMC7564340; doi:10.3390/cells9091995)
Supplement: Supplementary file 1 [file cells-09-01995-s001.pdf]

Supplementary Material

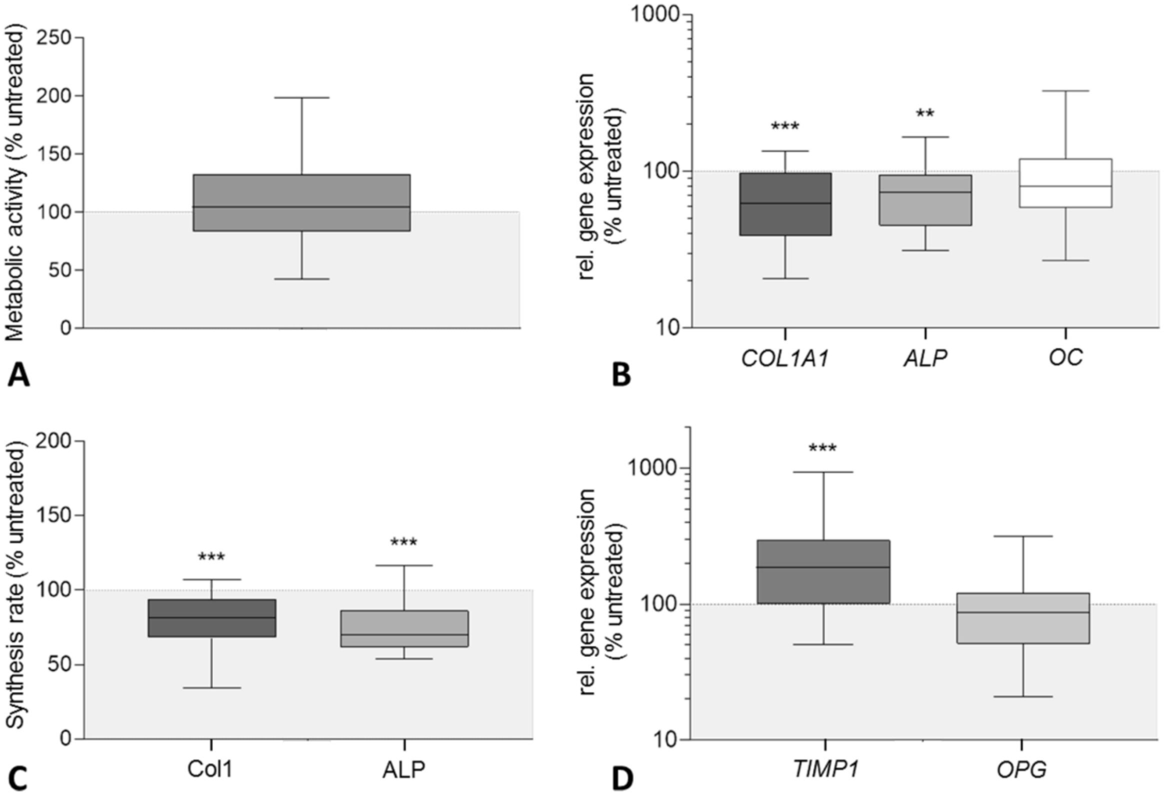

Figure S1. Influence of a static pressure load of 527 Pa conducted by titanium bodies [load] on osteoblastic viability (**A**) and expression of osteogenic markers (**B**–**D**). Human osteoblasts were seeded on collagen scaffolds and loaded with Ti6Al4V bodies for three days. Untreated cells served as control. A: The metabolic activity was determined colorimetrically by the conversion of tetrazolium salt to formazan (WST-1, n = 23). (**B**) Synthesis rates of procollagen type 1 and alkaline phosphatase (ALP) (untreated cells served as control). The release of procollagen type 1 propeptide in the cell culture supernatant was determined using ELISA and related to the total protein concentration. The ALP activity was determined colorimetrically by the hydrolysis of p-nitrophenyl phosphate (both: $n \geq 21$). (**C**,**D**): Relative gene expression of osteogenic markers for bone formation: collagen type I alpha 1 chain (COL1A1), alkaline phosphatase (ALP) and osteocalcin (OC) and bone remodeling: tissue inhibitor of metalloproteinase 1 (TIMP1) and osteoprotegerin (OPG). Gene expression of *COL1A1, ALP, OC* (all in Figure **B**), *TIMP1* and *OPG* (both in Figure **C**) was determined via semi-quantitative polymerase chain reaction (qPCR). Results are presented as the percentage of $2^{(-\Delta\Delta Ct)}$ related to the untreated controls (n = 23). All data are presented as box plots related to the untreated controls. ** $p <$ 0.01; *** $p < 0.001$ compared to the untreated controls (Wilcoxon’s signed-rank test).
